# A durian leaf image dataset of common diseases in Vietnam for agricultural diagnosis

**DOI:** 10.1016/j.dib.2025.111845

**Published:** 2025-06-30

**Authors:** Truong Nguyen Thanh, Linh Xuan Nguyen, Thang Cap, Tuong Le

**Affiliations:** a University of Information Technology, Vietnam National University, Ho Chi Minh City, Vietnam; b Faculty of Information Technology, HUTECH University, Ho Chi Minh City, Vietnam

**Keywords:** Durian leaf disease, Agricultural diagnosis, Computer vision in agriculture, Vietnam agriculture

## Abstract

Agriculture plays a vital role in Vietnam’s economy, with durian being a key high-value crop that supports millions of farmers. However, durian leaves are highly susceptible to pests, diseases, and environmental stressors, negatively impacting yield and quality. This study introduces a dataset of 2595 durian leaf images, categorized into six classes: 484 healthy leaves and 2111 diseased leaves spanning Blight (440), Colletotrichum (400), Algal (462), Phomopsis (411), and Rhizoctonia (398). The images were collected from durian orchards across Vietnam under diverse conditions, then background-removed, resized to 400 × 400 pixels, and manually annotated with expert guidance. This dataset provides a valuable resource for advancing research in automated plant disease detection, enabling the development of computer vision models for early diagnosis and precision farming, thereby supporting sustainable durian production and improved crop productivity.

Specifications Table

**Table:** 

Subject	Computer Sciences
Specific subject area	Deep Learning, Computer Vision, Image Processing, Image Classification
Type of data	Raw Images
Data collection	The iPhone 14 was used to take high-quality images of leaves on different days and under various lighting conditions and angles. The leaves were carefully examined for diseases, which were then classified and organized into corresponding folders based on each type of disease.
Data source location	The data was primarily collected from the two locations mentioned below. Location 1: Bu Dang District, Binh Phuoc Province, Vietnam Location 2: Cai Lay District, Tien Giang Province, Vietnam
Data accessibility	Repository: A Durian Leaf Image Dataset of Common Diseases in Vietnam for Agricultural Diagnosis Data identification number: 10.17632/pxzvksbwnj Direct URL to data: https://data.mendeley.com/datasets/pxzvksbwnj Instructions for accessing these data: The data is divided into three sets: train, test, and validation. Each set contains six folders with pre-processed images in JPG format.
Related research article	

## 1 Value of the Data

• The dataset consists of 2595 images of durian leaves captured via mobile devices, including healthy leaves and five common durian leaf diseases. The majority of the images were collected from Tien Giang and Binh Phuoc provinces in southern Vietnam. Furthermore, the dataset contains images taken under diverse environmental conditions, lighting scenarios, and shooting angles, ensuring a varied and representative collection for accurately classifying diseases in durian leaves.
• The diversity of the dataset is highly beneficial for training and testing deep learning models, particularly Convolutional Neural Networks (CNNs). Training significantly improves the performance and accuracy of disease detection and classification in durian leaves.
• Facilitates accurate and timely monitoring of diseased plants and enables early disease detection, helping to minimize crop losses and reduce dependence on chemical treatment methods.
• Valuable for the development of crop monitoring applications designed to assist farmers with effective crop health management, thereby enhancing agricultural product quality.

## 2 Background

The durian tree is a tropical fruit tree with high economic value and is widely cultivated in Southeast Asian countries [1]. In Vietnam, durian is primarily grown in the Mekong Delta, Southeastern, and Central Highlands regions, with a total plantation area of approximately 155,000 hectares. According to the General Statistics Office of Vietnam, in 2024, the country’s durian production reached 1,503.2 thousand tons, with an export value of $3.3 billion. This significant growth highlights the substantial development potential of durian cultivation, contributing to both domestic consumption and international market expansion.

However, durian cultivation faces numerous challenges in the context of climate change, including environmental factors and plant diseases [2]. Among these, durian leaf diseases pose a significant threat, directly affecting leaf growth and photosynthesis. Some common diseases include leaf blight (caused by Rhizoctonia solani), anthracnose (Colletotrichum zibethinum), and leaf spot (Phomopsis durionis) [3].

With the rapid development of artificial intelligence (AI), particularly computer vision, applications in agriculture are receiving increasing attention. AI-powered disease recognition and classification for durian leaves offer significant benefits. Computer vision models can automatically analyze images, detect diseases early, and accurately classify different types of infections based on leaf morphology. This reduces reliance on agricultural experts, increases the speed and accuracy of diagnosis, and optimizes disease management and farming processes.

Previous studies on durian leaf diseases have encountered considerable limitations. Specifically, the sample sizes in most studies range from only 500 to 1344 images, leading to suboptimal deep learning model performance. Additionally, many studies classify only four categories (three disease types and healthy leaves), whereas, in reality, durian leaf diseases can exhibit multiple variations [[4], [5], [6]]. Therefore, a comprehensive and diverse dataset is essential to reflect the actual conditions of durian leaf diseases accurately.

To address these limitations, our research has developed a new dataset comprising over 2595 images, categorized into six distinct disease classes: Durian Leaf Healthy (484 images), Durian Leaf Algal (462 images), Durian Leaf Phomopsis (411 images), Durian Leaf Colletotrichum (400 images), Durian Leaf Rhizoctonia (398 images), and Durian Leaf Blight (440 images). This dataset not only contains a larger sample size but also captures the diverse manifestations of each disease, enabling deep learning models to recognize complex and subtle patterns effectively. Consequently, AI-driven disease detection applications in agriculture will be significantly improved, supporting efficient disease monitoring and management for durian trees, enhancing productivity and product quality, and ultimately bringing substantial economic benefits to farmers.

## 3 Data Description

The dataset of durian leaf diseases was collected in January 2025 from Binh Phuoc and Tien Giang - two key durian-producing provinces. The dataset consists of 2595 images captured from various angles and under different weather conditions, including 484 images of healthy leaves and 2111 images of diseased leaves. The identified leaf diseases include anthracnose (Colletotrichum), algal spot (Algal), leaf spot (Phomopsis), blight and leaf wilt (Rhizoctonia), as well as other unidentified or miscellaneous leaf blight diseases.

**Fig. 1:**
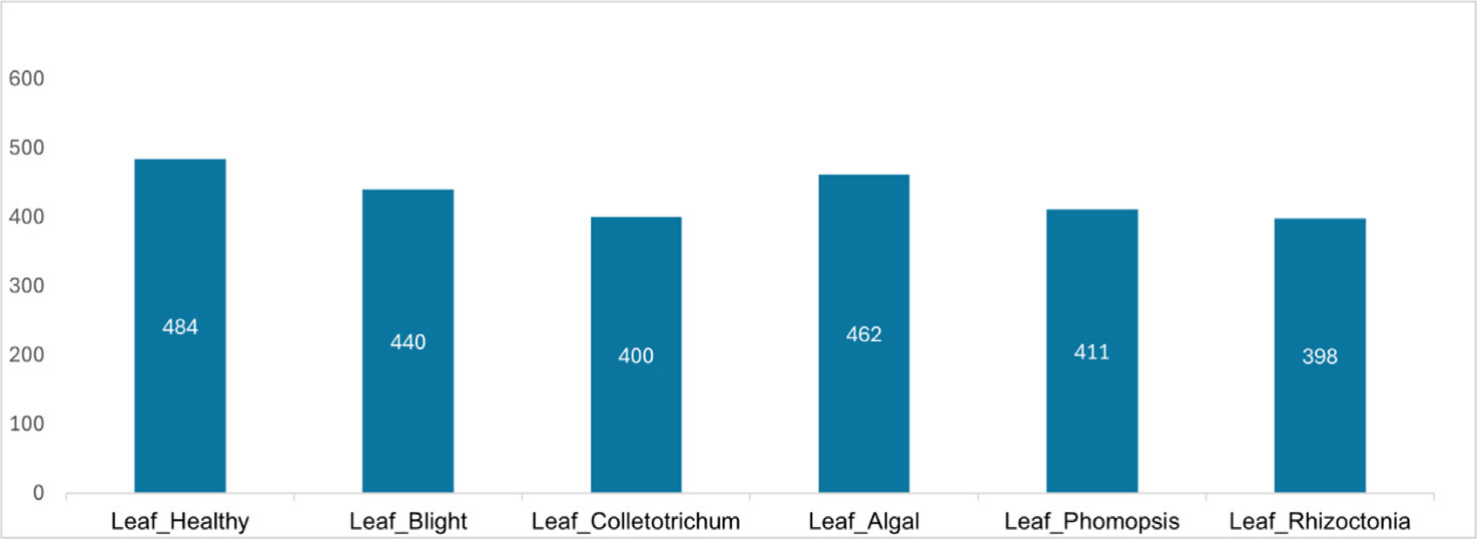
Distribution of durian leaf disease dataset.

The data is classified into six distinct categories: Leaf_Healthy (healthy leaves), Leaf_Algal, Leaf_Phomopsis, Leaf_Colletotrichum, Leaf_Rhizoctonia, and Leaf_Blight. Table 1 provides a detailed description and visualization examples for each of these categories. Overall, the dataset includes 484 images of healthy leaves and 2111 images of diseased leaves. The distribution of images across these six categories is illustrated in Fig. 1.

**Table 1:** Description of durian leaf diseases.

No	Class name	Description	Visualization
1	Leaf_Healthy	The leaf is vibrant green, intact, and unaffected by any harmful agents.	
2	Leaf_Algal	Affected leaves often exhibit brown, gray, or light green spots caused by algae.	
3	Leaf_Colletotrichum	The disease typically appears at the leaf tips or edges. The leaf blade turns dark brown, and characteristic lesions form concentric rings.	
4	Leaf_Phomopsis	On durian leaves, disease spots appear as pinhead-sized dots, each surrounded by a yellow halo. The lesions are oval-shaped, and in severe cases, they take on a crab-eye pattern with gray or brown discoloration along the central vein, gradually spreading inward.	
5	Leaf_Rhizoctonia	Water-soaked lesions appear on the leaves, later expanding and turning brown. Web-like fungal filaments spread across the leaf surface, forming clusters of infected areas.	
6	Leaf_Blight	Symptoms of leaf blight are caused by various factors or unidentified causes.

To enhance usability, the images were first cropped to isolate the characteristic features of the leaf diseases. Subsequently, they were all resized to a uniform dimension to ensure dataset standardization. This dataset accurately reflects the health conditions of durian leaves, providing a valuable resource for research and practical applications.

## 4 Experimental Design, Materials and Methods

**Fig. 2:**
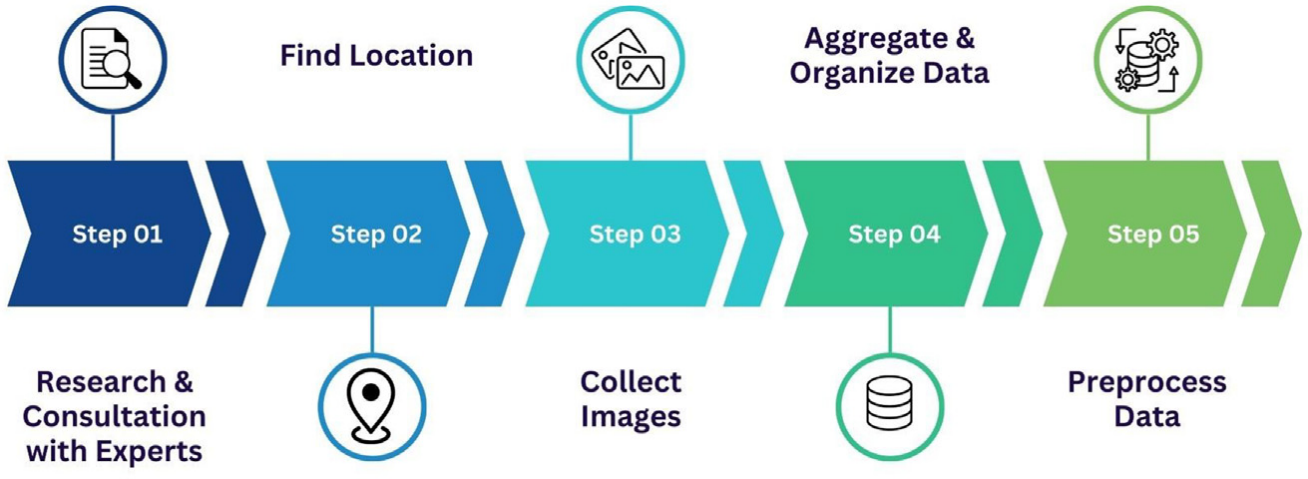
Illustration of preprocessing steps in the collection of diseased durian leaves.

Our project aims to build a specialized image dataset of durian leaf diseases to support research and the development of automated diagnostic tools, thereby contributing to crop productivity protection. To achieve this goal, we have established a structured data collection process based on field surveys. Fig. 2 illustrates the preprocessing steps involved in the collection of diseased durian leaves.

### 4.1 Literature Review and Expert Consultation

First, we conducted in-depth research on scientific and agricultural literature to identify common diseases affecting durian leaves. Since our team primarily comes from a computer science background, we made a dedicated effort to study plant biology and phytopathology to ensure accuracy during data collection and preliminary disease identification. Subsequently, to validate these initial identifications, we consulted with two agricultural experts. As part of a cross-checking process, the same set of collected durian leaf images was presented independently to each expert for verification. Notably, minimal disagreement was observed between the experts’ identifications, which we attribute in part to the thoroughness of our initial data collection and selection process. This expert consultation step, incorporating cross-checking, was crucial not only for confirming the accuracy of our disease assessments but also for gaining practical insights into disease characteristics.

### 4.2 On-Site Survey and Data Collection

Based on the gathered information, four durian orchards (two in Binh Phuoc and two in Tien Giang) were selected for on-site surveys. The surveyed orchards primarily cultivated the Monthong and Ri6 varieties, two dominant durian cultivars in Vietnam, known for their widespread commercial use. After presenting our objectives and receiving consent from orchard owners, we proceeded with image collection in January 2025. This process took place under various environmental conditions—ranging from hot sunny days (33-34°C) to rainy days (26-28°C)—and at different times of the day, including early morning (7-8 AM), noon (11 AM), and afternoon (3-4 PM). Collecting data at these time intervals helped capture the natural variations in plant conditions throughout the day. We used an iPhone 14 camera (12MP sensor, f/1.5, 26mm, OIS) to capture images from multiple angles and lighting conditions, optimizing data diversity. All images were then classified and stored in a structured format, ready for the preprocessing stage.

### 4.3 Image Annotation Process

Building on the foundation of expert consultation and addressing the need for a reproducible annotation protocol, we implemented a structured labeling process for disease classification using the Computer Vision Annotation Tool (CVAT). A new project was created within CVAT, defining the six distinct classes pertinent to this study: Leaf_Healthy, Leaf_Algal, Leaf_Phomopsis, Leaf_Colletotrichum, Leaf_Rhizoctonia, and Leaf_Blight. The raw images, exactly as captured by the camera without any prior processing (such as format conversion, renaming, background removal, or cropping), were uploaded directly to this platform.

Annotation Guidelines were developed using class descriptions and visual examples (Table 1), informed by expert input. Two trained annotators used these guidelines to assign a single image-level class label to each raw image. To rigorously assess the reliability of this classification process, an Inter-Annotator Agreement (IAA) analysis was conducted. Both annotators independently classified the same subset of approximately 10% of the data (260 images), which was randomly sampled from the full dataset using a standard random function with default parameters. The resulting agreement on the classification labels yielded a Percentage Agreement of 87.69% and a Cohen's Kappa (κ) score of 0.8516, indicating substantial agreement and confirming the clarity of the guidelines and the consistency between annotators. Following the successful IAA validation, the remaining 90% of the dataset was divided equally (50% each) between the two annotators for completion of the classification task.

For final quality control, a randomly selected 30% subset of the annotated dataset was reviewed by two agricultural experts, ensuring coverage across annotators and disease classes. Experts verified class labels against the guidelines. The verified annotations were exported from CVAT as mappings of image filenames to class labels. These were then reorganized into an ImageNet-style folder structure, with each folder corresponding to one of the six disease classes.

### 4.4 Data Preprocessing

Initially, images were captured using an iPhone in the HEIC format. Following the annotation process in CVAT, where each image was assigned a class label and subsequently organized into corresponding class-specific folders (as detailed in the Image Annotation Process section), several post-processing steps were undertaken. To ensure compatibility and consistency for model training, all images were then converted from their original HEIC format to JPG. Concurrently, the images were renamed according to the convention <disease_name>_<index>.JPG, utilizing the class name derived from their folder placement (determined during annotation).

The original dataset contained images with two common resolutions: 3628 × 2721 pixels and 3024 × 4032 pixels. To reduce background interference, we manually cropped the images to emphasize the primary durian leaf area, minimizing elements such as soil, branches, and unrelated foliage while preserving visible disease symptoms. Cropping was performed using consistent visual criteria to maintain uniformity across the dataset.

Subsequently, all images were transformed into square formats using centre cropping around the central diseased region. This technique preserved the spatial focus while avoiding geometric distortion commonly introduced by non-uniform scaling or stretching. Each square image was then resized to 400 × 400 pixels, a resolution empirically chosen to balance the trade-off between preserving visual details relevant to classification and reducing computational cost during training.

**Fig. 3:**
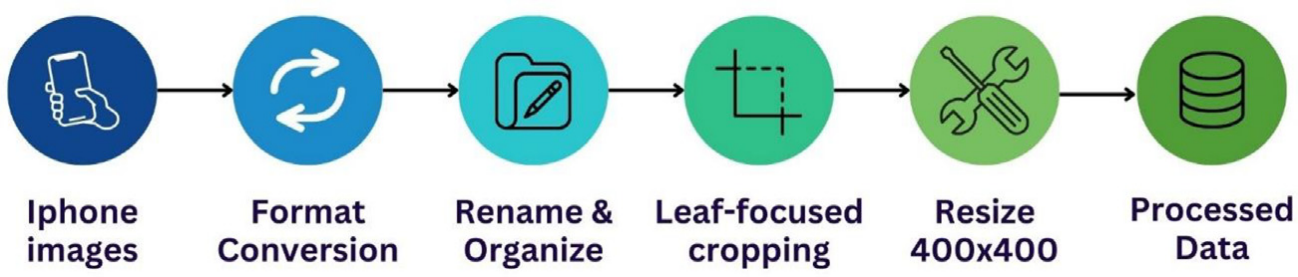
Data preprocessing workflow for diseased durian leaves.

This standardized preprocessing ensured consistent input dimensions, minimized distortion, and facilitated efficient batch processing for the deep learning model. The overall data processing pipeline is illustrated in Fig. 3.

After preprocessing, the dataset was divided into training, validation, and test sets using a proposed ratio of 70% (1814 images), 15% (387 images), and 15% (394 images), respectively, using class-stratified sampling. This method ensured proportional representation of all six classes—Leaf_Healthy, Leaf_Algal, Leaf_Phomopsis, Leaf_Colletotrichum, Leaf_Rhizoctonia, and Leaf_Blight—across each set, preventing class imbalance and supporting robust model training, validation, and evaluation. Table 2 summarizes the dataset partitioning.

**Table 2:** Proposed dataset split: training, validation, and test sets.

Dataset	Ratio (%)	Number of Images	Notes
Train	70	1814	Used for model training, requires a large portion to learn features.
Validation	15	387	Used to tune hyperparameters and evaluate during training.
Test	15	394	Used to assess final model performance, independent of training.

## Limitations

During the research process, we encountered various difficulties and challenges but managed to overcome them using fundamental methods. Below are some limitations of the study:

• **Limited geographical scope:** Data was collected only from two locations, Binh Phuoc and Tien Giang, leading to a potential regional bias. The environmental and soil conditions in these two areas may not fully reflect the biological diversity of durian trees nationwide, affecting the model’s generalization ability.
• **Restricted number of diseases**: The dataset includes only six common diseases, whereas, in reality, there may be many other diseases that have not been recorded. This limitation could reduce the model's accuracy when encountering cases outside the training dataset.
• **Diagnosis based on external symptoms**: Diseases were identified through direct observation of leaves without the support of specialized diagnostic equipment, such as molecular biology tests or histopathological analysis. If certain diseases show similar symptoms or are affected by other environmental factors, this can result in inaccurate diagnoses.

## Ethics Statement

All research procedures adhere to ethical principles. The study does not involve humans, animals, or data from social media. All data used in the research is publicly available, and we strictly follow proper citation guidelines.

## CRediT authorship contribution statement

**Truong Nguyen Thanh:** Writing – original draft, Data curation. **Linh Xuan Nguyen:** Writing – original draft, Data curation. **Thang Cap:** Supervision, Writing – review & editing. **Tuong Le:** Supervision, Writing – review & editing.

## Data Availability

Mendeley DataA Vietnamese Durian Leaf Disease Image Dataset for Agricultural Diagnosis (Original data).
